# Comparison of four outdoor mosquito trapping methods as potential replacements for human landing catches in western Kenya

**DOI:** 10.1186/s13071-021-04794-3

**Published:** 2021-06-12

**Authors:** Bernard Abong’o, John E. Gimnig, Bradley Longman, Tobias Odongo, Celestine Wekesa, Amos Webwile, Benjamin Oloo, Mercy Nduta, Margaret Muchoki, Diana Omoke, Daniel Wacira, Kevin Opondo, Eric Ochomo, Stephen Munga, Martin J. Donnelly, Richard M. Oxborough

**Affiliations:** 1 Centre for Global Health Research, Kenya Medical Research Institute, P.O. Box 1578, Kisumu, Kenya; 2 PMI VectorLink Project, Abt Associates Inc., Whitehouse, Milimani, Kisumu, Ojijo Oteko Road, P.O. Box 895-40123, Kisumu, Kenya; 3 Division of Parasitic Diseases and Malaria, Center for Global Health, Centers for Disease Control and Prevention, Atlanta, GA 30333 USA; 4 Liverpool School of Tropical Medicine, Pembroke Place, Liverpool, L3 5QA UK; 5 PMI VectorLink Project, Abt Associates Inc., 6130 Executive Blvd, Rockville, MD 20852 USA; 6 The United States President’s Malaria Initiative (PMI), US Embassy Nairobi, United Nations Avenue, Nairobi, Kenya

## Abstract

**Introduction:**

Longitudinal monitoring of outdoor-biting malaria vector populations is becoming increasingly important in understanding the dynamics of residual malaria transmission. However, the human landing catch (HLC), the gold standard for measuring human biting rates indoors and outdoors, is costly and raises ethical concerns related to increased risk of infectious bites among collectors. Consequently, routine data on outdoor-feeding mosquito populations are usually limited because of the lack of a scalable tool with similar sensitivity to outdoor HLC.

**Methodology:**

The *Anopheles* trapping sensitivity of four baited proxy outdoor trapping methods—Furvela tent trap (FTT), host decoy trap (HDT), mosquito electrocuting traps (MET) and outdoor CDC light traps (OLT)—was assessed relative to HLC in a 5 × 5 replicated Latin square conducted over 25 nights in two villages of western Kenya. Indoor CDC light trap (ILT) was run in one house in each of the compounds with outdoor traps, while additional non-Latin square indoor and outdoor HLC collections were performed in one of the study villages.

**Results:**

The MET, FTT, HDT and OLT sampled approximately 4.67, 7.58, 5.69 and 1.98 times more *An. arabiensis* compared to HLC, respectively, in Kakola Ombaka. Only FTT was more sensitive relative to HLC in sampling *An. funestus* in Kakola Ombaka (RR = 5.59, 95% CI 2.49–12.55, *P* < 0.001) and Masogo (RR = 4.38, 95% CI 1.62–11.80, *P* = 0.004) and in sampling *An. arabiensis* in Masogo (RR = 5.37, 95% CI 2.17–13.24, *P* < 0.001). OLT sampled significantly higher numbers of *An. coustani* in Kakola Ombaka (RR = 3.03, 95% CI 1.65–5.56, *P* < 0.001) and Masogo (RR = 2.88, 95% CI 1.15–7.22, *P* = 0.02) compared to HLC. OLT, HLC and MET sampled mostly *An. coustani*, FTT had similar proportions of *An. funestus* and *An. arabiensis*, while HDT sampled predominantly *An. arabiensis* in both villages. FTT showed close correlation with ILT in vector abundance for all three species at both collection sites.

**Conclusion:**

FTT and OLT are simple, easily scalable traps and are potential replacements for HLC in outdoor sampling of *Anopheles* mosquitoes. However, the FTT closely mirrored indoor CDC light trap in mosquito indices and therefore may be more of an indoor mimic than a true outdoor collection tool. HDT and MET show potential for sampling outdoor host-seeking mosquitoes. However, the traps as currently designed may not be feasible for large-scale, longitudinal entomological monitoring. Therefore, the baited outdoor CDC light trap may be the most appropriate tool currently available for assessment of outdoor-biting and malaria transmission risk.

**Graphic abstract:**

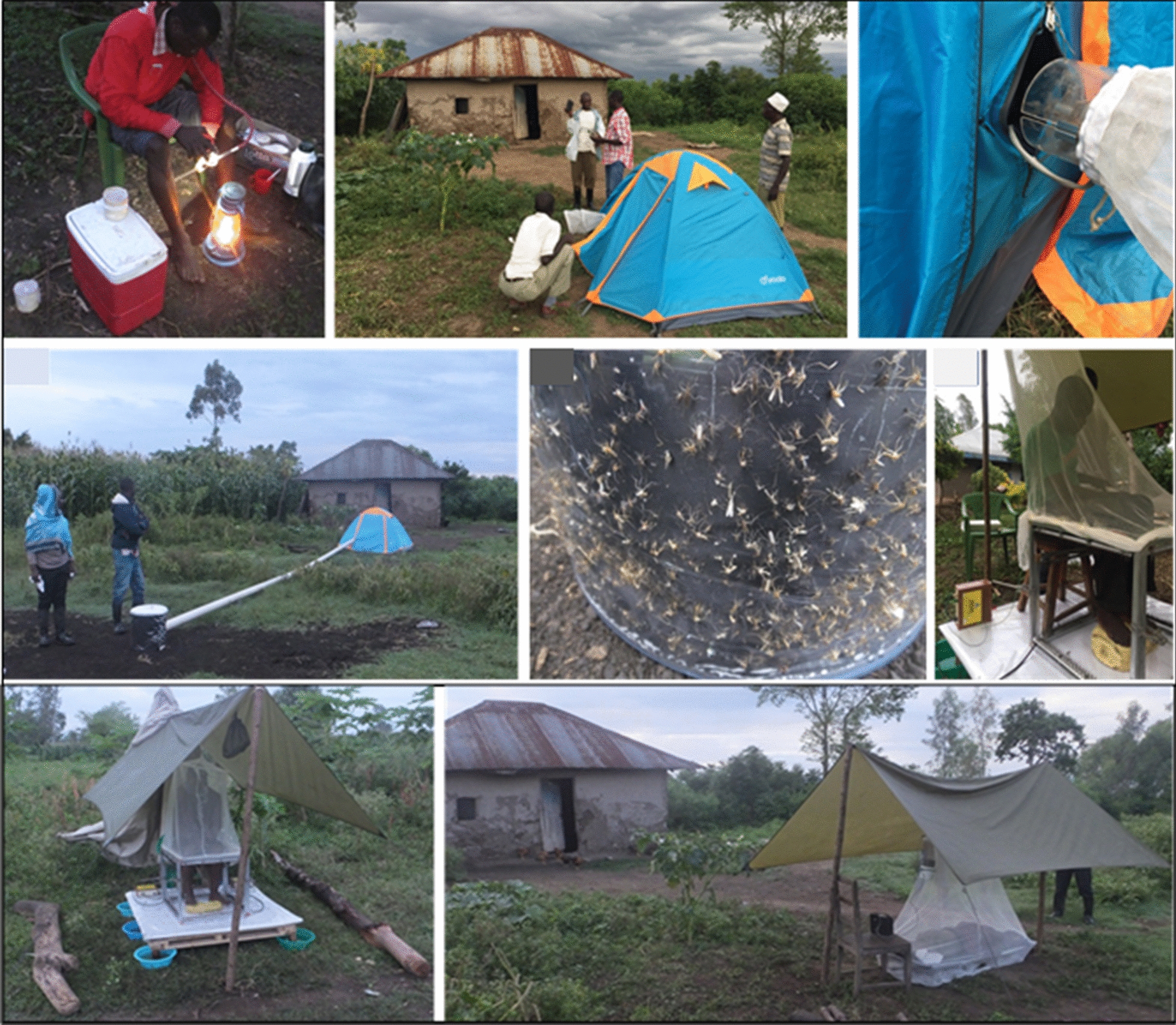

**Supplementary Information:**

The online version contains supplementary material available at 10.1186/s13071-021-04794-3.

## Introduction

*Anopheles funestus* and *An. gambiae*/*An. coluzzii* are considered to be the most efficient malaria vectors in sub-Saharan Africa, largely due to their high levels of anthropophagy [1]. The endophagic and endophilic nature of these primary malaria vector species has led to the use of widespread indoor focused interventions including insecticide treated nets (ITNs) and indoor residual spraying (IRS). The implementation of these measures has contributed to substantial reductions in the malaria burden in sub-Saharan Africa since 2000 [2, 3]. However, sustained indoor targeted control measures have been associated with changes in malaria vector species composition [4–6] and possibly behaviour [7–9]. In some settings, these changes have been associated with increased proportions of more exophilic vector species such as *An. arabiensis* or changes in the biting and resting behaviour of *An. gambiae* or *An. funestus*, leading to relative increases in outdoor transmission [10–13]. Sustained indoor vector control through ITNs and IRS in western Kenya has resulted in changes in species composition, with the more generalist species *An. arabiensis* becoming the predominant species in some locations [4, 7] and changes in *An. gambiae* (*s.s.*) behaviour resulting in earlier biting [9] with a high frequency of animal and mixed human-animal blood meals [8]. Longitudinal monitoring of vector species composition, biting rates, physiological status, biting and resting behaviour, and ecology is fundamental to understanding the risk of malaria transmission in an area, identifying future threats and formulating methods of control and monitoring [14]. However, outdoor biting mosquitoes pose new challenges in sub-Saharan Africa as they cannot be reliably monitored using many of the current collection methods and tools to control them effectively are lacking.

When conducted under close supervision, the human landing catch (HLC) is generally considered the gold standard for indoor and outdoor collections which can provide critical information on malaria vector species composition, biting densities and time of biting [15]. However, HLC is a labour-intensive and costly procedure requiring trained collectors and extensive supervision [16, 17] and is unsustainable for large-scale operational sampling of malaria vectors. There are also ethical questions regarding the use of human collectors to attract pathogen transmitting mosquitoes [18, 19]. While provision of malaria chemoprophylaxis has been demonstrated to be protective to HLC collectors [20], there is still potential for transmission of arboviruses and other mosquito-transmitted pathogens [18]. Due to these ethical concerns coupled with logistical challenges in HLC implementation, the technique is rarely used for routine monitoring of mosquito populations.

Routine monitoring of indoor mosquito populations in western Kenya has been conducted using indoor Centers for Disease Control and Prevention light trap (ILT) and pyrethrum spray catch (PSC). The ILT has been observed by some studies to be an effective alternative to indoor HLC [21–24], whereas other studies have reported the trap to collect fewer *Anopheles* than HLC [25]. Mathenge et al. (2005) showed that ILT collected 60% of *An. arabiensis* and 120% of *An. funestus* compared to indoor HLC [24]. Pyrethrum spray catch (PSC) and ILT have become standard trapping methods to monitor indoor populations, but no reliable method has emerged to replace HLC outdoors. As a result, longitudinal entomological monitoring conducted by the President’s Malaria Initiative (PMI) VectorLink Project from 2017 to 2018 consisted only of indoor trapping using ILT, PSC and use of window exit traps, with no routine outdoor trapping taking place.

Scalable traps designed specifically for monitoring outdoor biting and resting malaria vectors are urgently needed [12]. The choice of collection method for operational surveillance should be driven by trap efficacy and scalability rather than fine-scale precision with respect to human landing catches (HLC) [23]. This study compared four outdoor traps with HLC as a positive control to identify a suitable replacement for the HLC for longitudinal surveillance of outdoor biting malaria vectors. The primary objective was to determine outdoor trap efficacy for estimating *Anopheles* numbers per trapping night in comparison with the ‘gold standard’ HLC. Secondary objectives were to determine the outdoor biting mosquito species composition and to determine endophily proportion by comparing each outdoor trap with indoor CDC light trap (ILT).

## Methods

### Study sites

**Fig. 1:**
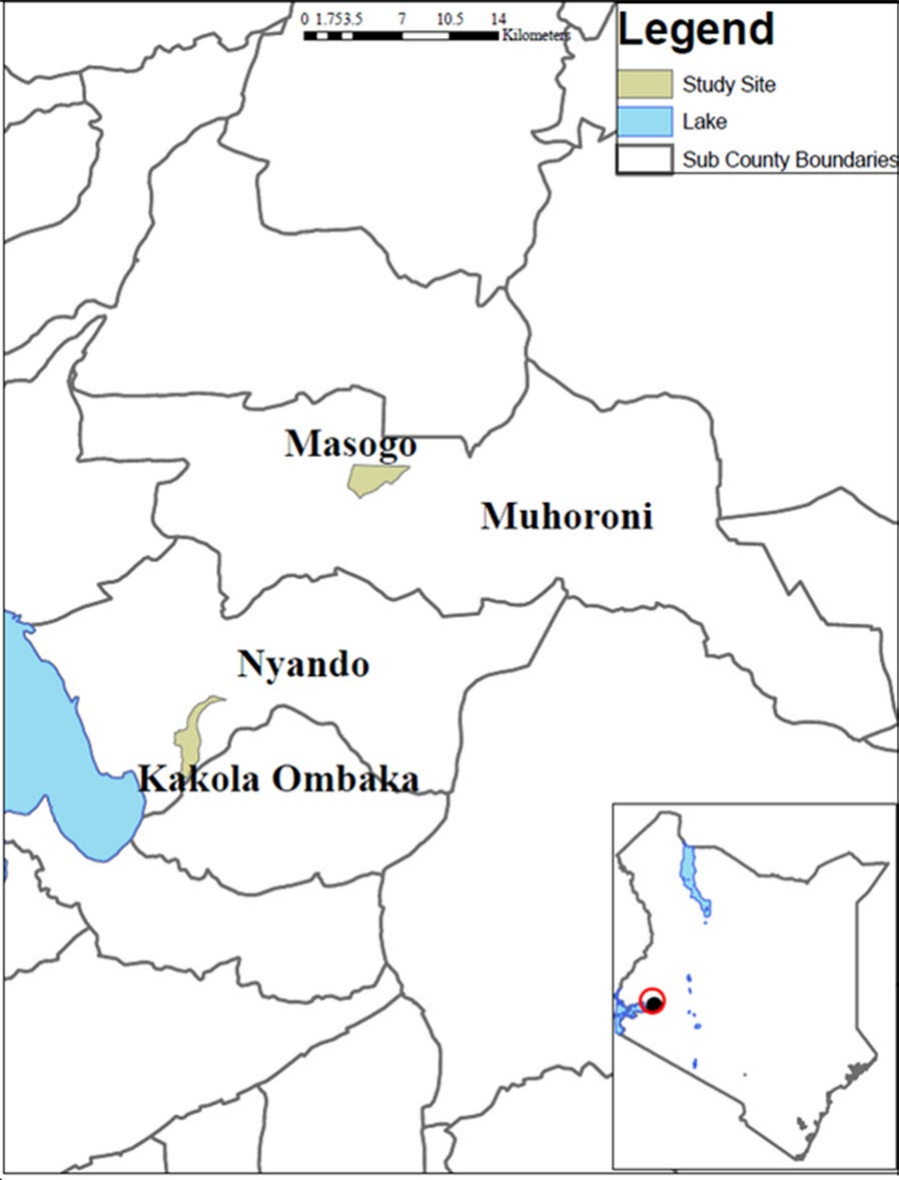
Map of the study area

The study was conducted in May/June 2018 in two villages, Kakola Ombaka (0.25°S, 34.88°E) near the Ahero rice irrigation scheme in Nyando sub-county and Masogo village (0.16° S, 35.19° E) in Mhoroni sub-county, Kisumu County, western Kenya (Fig. 1). The villages were selected to represent areas with high (Kakola Ombaka) and low (Masogo) mosquito densities. The study was conducted in the months of May and June coinciding with the end of the main rainy season and the period of peak mosquito numbers and malaria transmission.

### Outdoor trapping methods

**Fig. 2:**
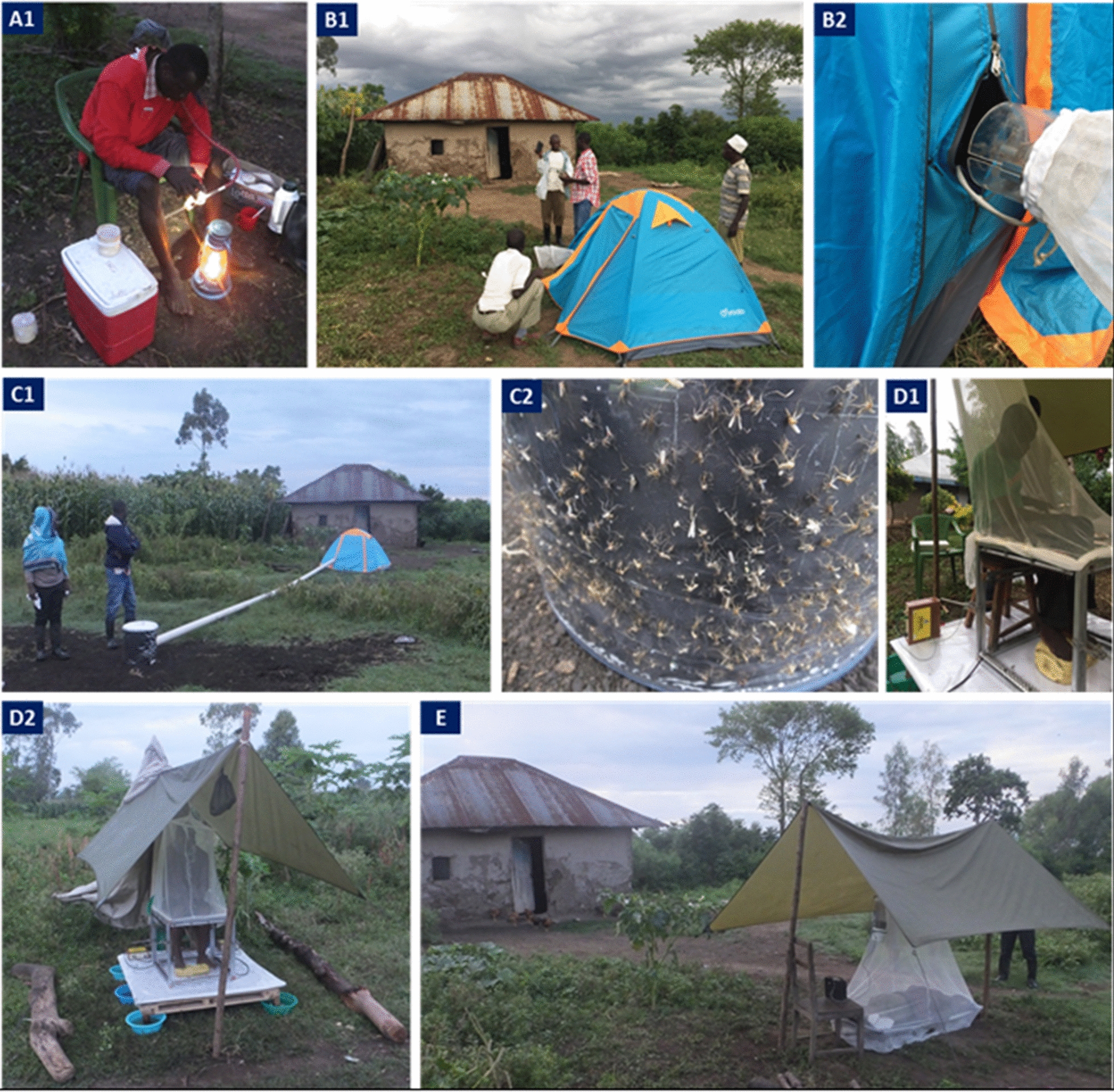
Photographs showing the five outdoor trapping methods that were compared. **A1** Human landing catch (HLC), **B1** Furvela tent trap (FTT), **B2** FTT opening with CDC light trap (light removed) attached, **C1** human decoy trap (HDT), showing tube taking human odour to the heated cylinder sticky trap, **C2** HDT, showing mosquitoes stuck to sticky panel on heated cylinder, **D1**, **2** electrocuting grid trap (EGT), **E** outdoor CDC light trap (CDC LT), hung next to human sleeping inside an untreated bed net

Four trapping methods—Furvela tent-trap Mk 1.1 (FTT), host decoy trap (HDT), mosquito electrocuting trap (MET) and outdoor CDC-LT (OLT)—were compared with the human landing catch (HLC) in a 5 × 5 replicated Latin square experimental design of trapping methods × housing compounds (Additional file 1: Table S1). The study was conducted for 25 nights in five compounds per night in each village. Distance between the trapping compounds was approximately 100 m. Traps were rotated between the housing compounds nightly, so that over five trapping nights each trap was rotated between all five house compounds. Collectors worked in two shifts each night and were rotated weekly between collection locations each night. Housing compounds where outdoor traps were set were at least 100 m from each other to minimize the risk of host-odour interference. The location for each outdoor collection method was marked in each compound to ensure consistency throughout the study. Outdoor collections were made approximately 5–10 m away from the house in a cleared space. In each housing compound where outdoor trapping was conducted, an indoor CDC light trap (ILT) was set inside the house each night for comparison with the outdoor catch. The ILT was installed in a bedroom at approximately 1.5 m above the floor next to an occupied *in situ* ITN. All collections were performed from 18:00 to 07:00 the following morning. Figure 2 shows photographs of the five outdoor trapping methods. These are described in more detail below:

### Furvela tent trap

The Furvela tent trap was developed and tested in the village of Furvela, Mozambique, and has been utilized in several locations including Tanzania, Ghana and Cambodia [26–28]. The basic principle of the Furvela tent trap is that human odour and exhaled gases emanating from a gap, the diameter of a CDC trap, in the predominantly closed door of the tent, attract mosquitoes to the gap on the tent door. Close to the gap, the fan and collection bag of a CDC trap (without the light, lid or grid) are placed horizontally outside the tent, 2 to 3 cm from the opening in the door. On approach to the opening, the insects are sucked into the trap and held in the trap collection bag (Fig. 2B1–2). The suction from the fan effectively prevents any mosquitoes from entering the tent, even at high densities, so that the sleeper is not exposed to biting risk. Mosquitoes are removed from the collection bag using a mouth aspirator and transferred to a holding cup.

### Host decoy trap

The Host Decoy Trap (HDT) was first evaluated in the field in 2015 in Burkina Faso. The HDT exploits the blood-seeking behaviour of mosquitoes by mimicking the sensory stimuli that a mosquito follows when searching for a person to bite. These include host odour, a visual stimulus and body temperature of warm-blooded hosts. These stimuli are incorporated into a trap that lures mosquitoes towards it and then captures them when they land. The trap was set as described by Hawkes et al. [29]. Briefly, the host decoy trap is a cylindrical container filled with warm water, insulated with Styrofoam to prevent heat loss and regulate the surface temperature. The container is covered with a black jacket to provide visual contrast and transparent sticky tape to which mosquitoes get stuck on landing. Host odour from a nearby occupied tent is exhausted using a fan, pushed through a pipe and vented close to the trap (Fig. 2C1-2). Mosquitoes attracted to an odour source are induced to land upon the visually conspicuous, warm trap, where upon they get stuck. The mosquitoes are recovered from the trap by dissolving the glue upon which they are stuck [29].

### Electrocuting grids

These devices were originally developed to quantify the numbers of tsetse flies attracted to humans and wildlife hosts by placing electrocuting nets in an incomplete ring around the host species [30]. The electrocuting grid is effectively invisible to flying insects which, as they approach a stimulus such as a vertebrate host, inadvertently collide with it and are either killed or stunned. Electrocuting grids (0.5 m high; 1 m wide) consisted of vertical copper wires, 0.2 mm in diameter, 5 mm apart and spray painted black to reduce their visibility to flying insects. Alternate wires were earthed or charged by a transformer with a direct current (DC) input (12 V: 3 A) and an output of 50 kV, pulsing at ~ 70 Hz. Insects killed or stunned after colliding with the grids were collected on a sticky panel placed under the electrocuting grid. A simple shelter was erected over the collector to protect them from the rain. The collector sat on a stool and four panels of electrocuting grids were arranged around the legs up to the knee level. The rest of the body was covered with an untreated bed net attached to the top frame of the electrocuting grid. Mosquitoes attempting to access the collector were electrocuted and dropped on the sticky panel under the grid from where they were collected and recovered by dissolving the glue (Fig. 2D1–2).

### CDC light trap (outdoors)

CDC light traps are battery powered with a motorized fan, light bulb and a mosquito collection cup. The trap can be used with CO_2_ to mimic breath exhalation. Mosquitoes attracted to the traps by either light and/or CO_2_ are drawn in at the top and forced downward by the fan into the collection cup, from which they cannot escape. Malaria-transmitting mosquitoes are nocturnal; therefore, traps are typically deployed at dusk and collected at dawn the following day. When deployed indoors, the optimum location for sampling house-visiting mosquitoes has been reported to be as close as possible to the host, with improved catching efficiency when the trap is installed at the foot of an occupied untreated bednet [31, 32]. From an epidemiological point of view, the use of a light trap next to an occupied bednet is more meaningful than using an unbaited light trap [33] as it provides a good measure of host-seeking mosquito densities. Outdoor deployment of light traps is not commonly used in surveillance particularly in regions where people are mostly indoors at night. The CDC-LT was hung outdoors at 1.5 m above the ground next to an occupied, untreated bed net (Fig. 2E). The bed and light trap were protected from rainfall by a tarpaulin.

### Human landing catch (outdoors)

**Fig. 3:**
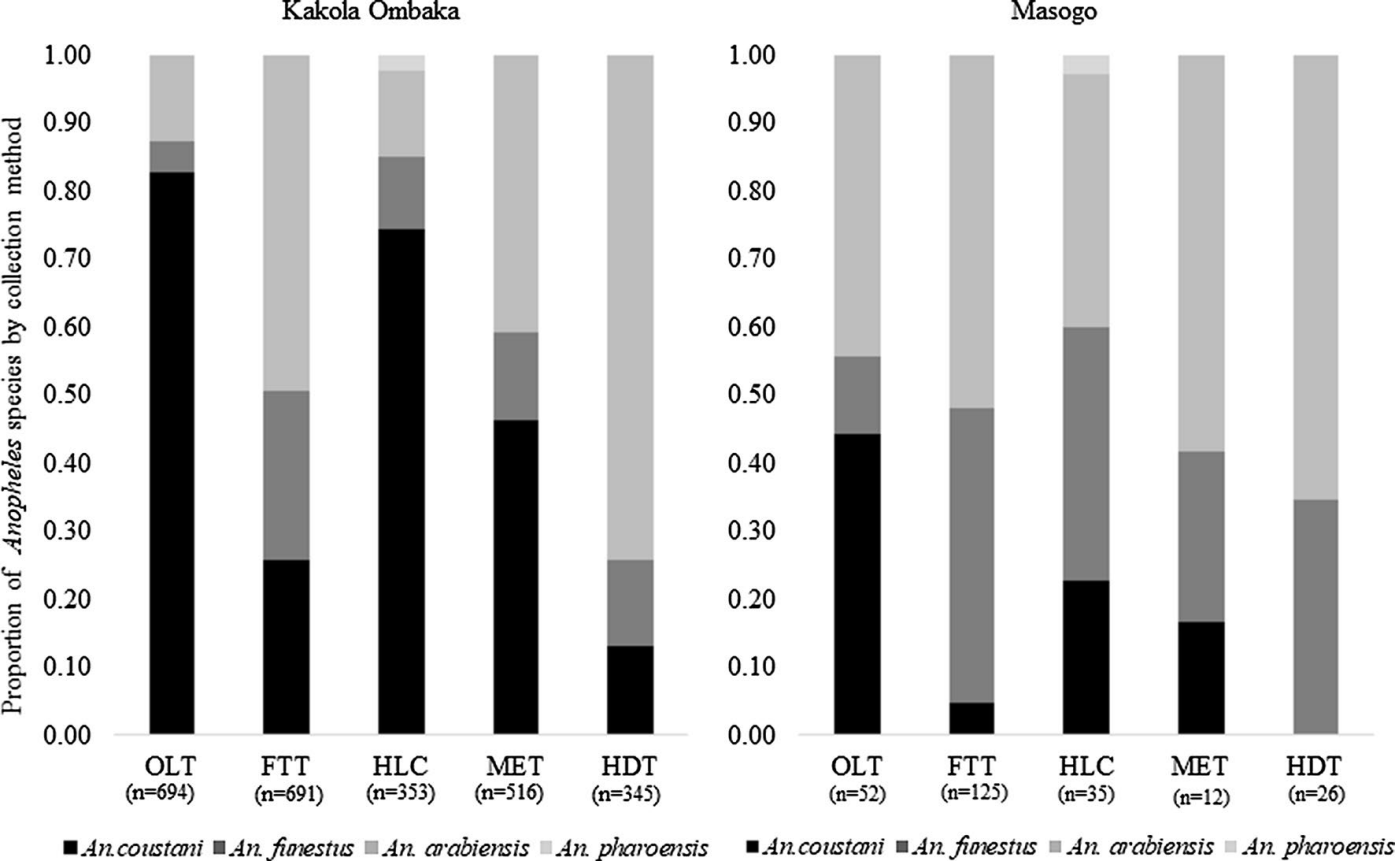
Relative species composition (proportions) of *Anopheles* mosquitoes from MET, FTT, HDT, OLT and HLC in Kakola Ombaka and Masogo villages over the study period in western Kenya

One collector sat outside on a chair with lower limbs exposed and a dimly lit kerosene lamp nearby to provide some light. A torch (flashlight) was used to spot the mosquitoes landing on the exposed lower limbs of the HLC collector. Mosquitoes landing on the exposed limbs were aspirated and transferred into paper cups labelled with the hour of collection (Fig. 3, panel A1). Within each collection hour, the collectors worked for 45 min with 15 min breaks between the hours and switched shifts at midnight. Collectors provided written consent to participate in the study. They were tested for malaria infection using a malaria rapid diagnostic test 7 days before collections began and all tested negative. The collectors were placed on weekly malaria prophylaxis beginning 7 days before collections began and continuing up to 4 weeks after the end of collections. Over the same period, the collectors were monitored for malaria infection. None tested positive during and up to 4 weeks after HLCs were conducted. Each collector was compensated for time spent in mosquito collection.

### Additional non-Latin square human landing catch

To assess malaria vector biting behaviour, *Plasmodium falciparum* infection rates and risk of human to exposure to mosquito bites, an additional 5 nights of HLCs were conducted indoors and outdoors in five houses in Kakola Ombaka. The collections were performed in the last of week of the Latin square outdoor trap comparison study. In each collection house, four collectors worked in pairs in two shifts over the collection period. In each shift, one of the collectors worked outdoors within 5 m of the house while the other was indoors in the unoccupied living room. The collectors were rotated between the collection shifts and location. The HLC collectors also recorded the location of household members (indoors or outdoors) every hour to assess their risk of exposure to mosquito bites.

#### Laboratory analysis

All collected *Anopheles* were identified morphologically using the keys of Gillies and Coetzee, 1987 [34]. Further species discrimination was performed only for *An. gambiae* (*s.l.*) and *An. funestus* groups while all females were analysed for sporozoite infection. Female mosquitoes were dissected into parts for various procedures: heads and thoraces were used for determination of *P. falciparum* sporozoite infection by enzyme-linked immunosorbent assay (ELISA) using standard operating procedures as described in the MR4 Methods in *Anopheles* Research [35], adapted from Wirtz et al. [36]. The legs and wings were used in PCR analyses to identify to species level members of the *An. gambiae* species complex and *Anopheles funestus* group [35]. The protocol of Scott et al. [37] as described in standard operating procedures in the MR4 Methods in *Anopheles* Research [35] was used for distinguishing between different species of *An. gambiae* while the protocol of Koekemoer et al. [38] was used to identify members of the *An. funestus* species group.

#### Data analysis

Analysis was done using R statistical software version 4.0.2. Data were fitted using Generalized Linear Mixed Effects Models (GLMMs) to describe how well the other traps proportionately sample the same composition and species as HLC. Since the data were over-dispersed, the package glmmADMB [39] was used to fit negative binomial distribution models for the analysis of mosquito numbers in the outdoor traps for the Latin square study. The numbers of female *Anopheles* mosquitoes were assessed as a function of collection method as a fixed effect while collection compounds and days were treated as random factors. The same statistical package was used to assess catch sizes in indoor CDC light traps and each of the paired outdoor traps; the numbers of female *Anopheles* mosquitoes were assessed as a function of collection method as a fixed effect and collection compound as a random factor. Pairwise comparisons of the mean numbers of each *Anopheles* species collected by the different trapping methods were done by Tukey’s test. To analyse *Anopheles* species proportions for each trapping method, a binomial GLM model was used.

## Results

### Mosquito species composition and abundance

From the Latin square comparison of outdoor traps, a total of 2849 female *Anopheles* mosquitoes and 20,093 *Culex* species were collected outdoors at five sampling locations in each of the two sites over 25 trapping nights outdoors. The most abundant of the *Anopheles* species based on morphological identification was *An. coustani* (1339, 47.0% of all *Anopheles* mosquitoes) followed by *An. gambiae* (*s.l.*) (1066, 37.4%), *An. funestus* group (435, 15.3%) and *An. pharoensis* (9, 0.3%). Only 11 male *Anopheles* mosquitoes were sampled over the collection period (Table 1). Of the female *An. gambiae* (*s.l.*) from outdoor traps, 563 samples were analysed by PCR for species identification, and of these, 557 (98.9%) were *An. arabiensis* and 6 (1.1%) *An. gambiae* (*s.s.*). Of 212 samples morphologically identified as *An. funestus* group, all were identified by PCR as *An. funestus* (*s.s.*).

**Table 1:** Numbers of *Anopheles* and *Culex* mosquitoes sampled by different collection methods outdoor (Latin square study) and indoor by CDC light traps in Kakola Ombaka and Masogo villages over 25 trapping nights

Collection location	Study site	Collection method	Female *Anopheles*				Total female *Anopheles*	Male *Anopheles*	Total female *Culex* species
			*An. funestus*	*An. arabiensis*	*An. coustani*	*An. pharoensis*			
Outdoor	Kakola Ombaka	MET	67	210	239	0	516	2	2845
		FTT	172	341	178	0	691	2	4434
		HDT	44	256	45	0	345	1	2666
		OLT	30	89	575	0	694	3	3712
		HLC	37	45	263	8	353	1	3808
	Masogo	MET	3	7	2	0	12	1	409
		FTT	54	65	6	0	125	0	713
		HDT	9	17	0	0	26	0	411
		OLT	6	23	23	0	52	0	236
		HLC	13	13	8	1	35	1	859
	**Total**		**435**	**1066**	**1339**	**9**	**2849**	**11**	**20,093**
Indoor (ILT)	Kakola Ombaka	ILT	779	1176	954	3	2912	53	6157
	Masogo	ILT	157	167	13	0	337	44	1393
	**Total**		**936**	**1343**	**967**	**3**	**3249**	**97**	**7550**

Collections by indoor CDC light trap at each of the five trapping locations in the two sites yielded a total of 3249 *Anopheles* mosquitoes and 7550 *Culex* species. Among the *Anopheles* mosquitoes, 1343 were *An. gambiae* (*s.l.*) (41.3% of the total *Anopheles*), 967 were *An. coustani* (29.8%), 936 were *An. funestus* group (28.8%) and 3 were other *Anopheles* species (0.1%) (Table 1). Of 958 *An. gambiae* (*s.l.*) collected by indoor CDC light trap and identified to species by PCR, *An. arabiensis* was predominant (99.0%) with only 10 identified as *An. gambaie* (*s.s.*) (1.0%). Five hundred thirty-three (533) from *An. funestus* group were analysed by PCR and all were confirmed to be *An. funestus* (*s.s.*). Hereafter, all *An. gambiae* (*s.l.*) are assumed to be *An. arabiensis* and all *An. funestus* (*s.l.*) to be *An. funestus* (*s.s.*).

Figure 3 shows percentage of *Anopheles* species composition by trapping method for both the Kakola Ombaka and Masogo sites. The *Anopheles* species composition differed by trapping method in both sites. *Anopheles coustani* was the predominant species collected by OLT and HLC in Kakola Ombaka village, accounting for 82.9% of *Anopheles* by OLT and 74.5% by HLC. *An. arabiensis* was predominant in HDT and FTT, accounting for 74.2% of *Anopheles* in HDT and 49.4% in FTT. *Anopheles funestus* was collected in lower proportions in all outdoor traps compared to *An. coustani* and *An. arabiensis*. In Masogo village, *An. arabiensis* was the most common species across all collection methods: 44.2% of *Anopheles* mosquitoes in OLT, 52.0% FTT, 37.1% HLC, 58.3% MET and 65.4% HDT. *An. pharoensis* were only observed in HLC at both sites, representing 2.3% of all *Anopheles* collected in Kakola Ombaka and 2.9% in Masogo.

### *Anopheles* densities according to trap method

**Fig. 4:**
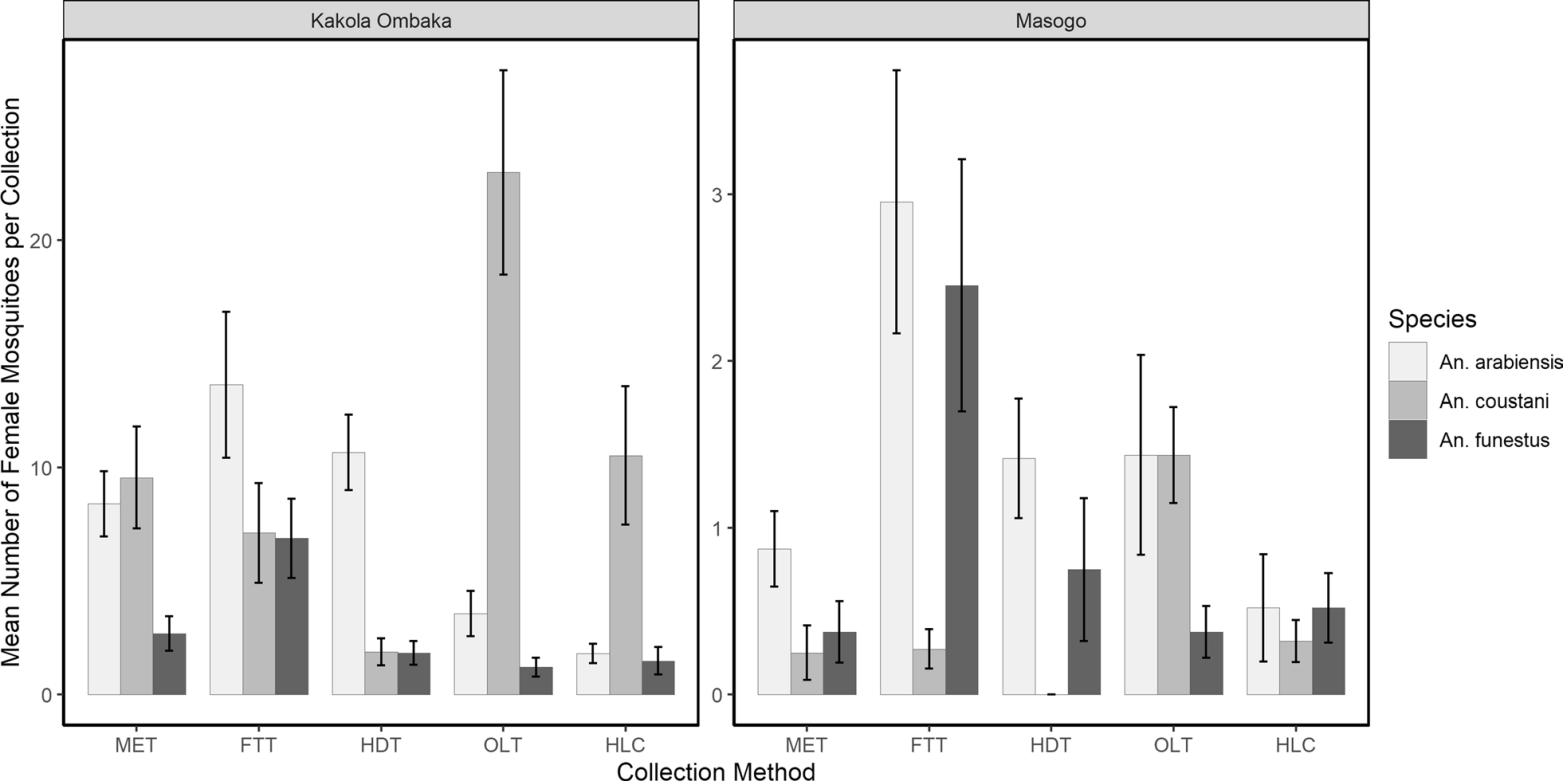
Nightly outdoor catches (mean ± SE) of *Anopheles* spp. from MET, FTT, HDT, OLT and HLC in Kakola Ombaka and Masogo villages. The graphs are of different scales

The mean numbers of *Anopheles* species collected outdoors by MET, FTT, HDT, OLT and HLC at Kakola Ombaka and Masogo villages are presented in Fig. 4 and Table 2. The mean number of *An. funestus* per trapping night was significantly higher in FTT compared to HLC in both Kakola Ombaka (RR = 5.59, 95% CI 2.49–12.55, *P* < 0.001) and Masogo (RR = 4.38, 95% CI 1.62–11.80, *P* = 0.004). Significantly fewer *An. funestus* were collected by MET (0.12) compared to HLC (0.52) in Masogo (RR = 0.24, 95% CI 0.05–1.03, *P* = 0.05). For all other traps there was no significant difference in the mean nightly catch of *An. funestus* between MET, HDT, OLT and HLC in Kakola Ombaka or between HDT, OLT and HLC in Masogo village.

**Table 2:** Comparison of mean numbers of *An. funestus*, *An. arabiensis* and *An. coustani* collected outdoors by MET, FTT, HDT, OLT and HLC in Kakola Ombaka and Masogo villages over the study period

Category	Collection Method	Kakola Ombaka					Masogo				
		Mean	Risk ratio	Lower CL	Upper CL	*P*-value	Mean	Risk ratio	Lower CL	Upper CL	*P*-value
*An. funestus*	MET	2.68	1.98	0.88	4.46	0.1	0.12	0.24	0.05	1.03	**0.05**
	FTT	6.88	5.59	2.49	12.55	** < 0.001**	2.16	4.38	1.62	11.8	**0.004**
	HDT	1.76	1.38	0.60	3.18	0.44	0.36	0.66	0.21	2.09	0.48
	OLT	1.20	0.88	0.37	2.11	0.78	0.24	0.45	0.13	1.57	0.21
	HLC	1.48	Ref				0.52	Ref			
*An. arabiensis*	MET	8.40	4.67	2.44	8.93	** < 0.001**	0.28	0.59	0.19	1.87	0.37
	FTT	13.64	7.58	3.98	14.42	** < 0.001**	2.26	5.37	2.17	13.24	** < 0.001**
	HDT	10.24	5.69	2.98	10.86	** < 0.001**	0.68	1.32	0.49	3.59	0.58
	OLT	3.56	1.98	1.01	3.86	**0.05**	0.92	1.83	0.70	4.79	0.22
	HLC	1.80	Ref				0.52	Ref			
*An. coustani*	MET	9.56	1.11	0.61	2.03	0.74	0.08	0.00	0.00	Inf	1.00
	FTT	7.12	0.66	0.35	1.23	0.19	0.24	7.50	0.24	2.37	0.62
	HDT	1.80	0.18	0.09	0.37	** < 0.001**	0.00	0.00	0.00	Inf	1.00
	OLT	23.00	3.03	1.65	5.56	** < 0.001**	0.92	2.88	1.15	7.22	**0.02**
	HLC	10.52	Ref				0.32	Ref			

The models included terms for collection methods and an interaction term. The risk ratios (RR) were generated by exponentiating the model coefficients

Significantly more *An. arabiensis* were collected by all the outdoor collection methods compared to HLC in Kakola Ombaka: MET (RR = 4.67, 95% CI 2.44–8.93, *P* < 0.001), FTT (RR = 7.58, 95% CI3.98–14.42, *P* < 0.001), HDT (RR = 5.69, 95% CI 2.98–10.86, *P* < 0.001) and OLT (RR = 1.98, 95% CI 1.01–3.86, *P* = 0.05). However, in Masogo, significantly higher numbers of *An. arabiensis* were only observed in FTT compared to HLC (RR = 5.37, 95% CI 2.17–13.24, *P* < 0.001). No significant differences were observed in the catch sizes of *An. arabiensis* between HLC and any of the other collection methods in Masogo.

Significantly higher numbers of *An. coustani* were caught by OLT compared to HLC in Kakola Ombaka (RR = 3.03, 95% CI 1.65–5.56, *P* < 0.001) and in Masogo village (RR = 2.88, 95% CI 1.15–7.22, *P* = 0.02) (Table1). There were significantly fewer *An. coustani* caught by HDT compared to HLC (RR = 0.18, 95% CI 0.09–0.37, *P* < 0.001) in Kakola Ombaka. For all other trapping methods there was no significant difference in *An. coustani* mean collection densities. Pairwise comparisons of mean densities of different *Anopheles* species by collection methods in Kakola Ombaka and Masogo are presented in Additional file 1: Tables S2, S3 respectively.

### Comparison of outdoor traps with indoor CDC light trap

We compared each of the outdoor traps with the paired indoor light trap data to assess catch size ratios of *An. arabiensis*, *An. funestus* and *An. coustani* indoors and outdoors in the same house compound (Tables 3 and 4). This comparison is useful in determining the endophilic/exophilic nature of each species in the area and determining the relative performance of each outdoor trap.

**Table 3:** Comparison of mean numbers of *An. funestus*, *An. arabiensis* and *An. coustani* collected outdoors and indoors between each of the outdoor trapping methods (MET, FTT, HDT, OLT, HLC) and ILT in Kakola Ombaka village

Comparison	*Anopheles* species	Collection method	Mean	Risk ratio	Lower CL	Upper CL	*P*-values
MET and ILT	*An. arabiensis*	MET	8.40	0.97	0.42	2.29	0.96
		ILT	13.08	Ref			
	*An. funestus*	MET	2.68	0.51	0.24	1.11	0.09
		ILT	5.28	Ref			
	*An. coustani*	MET	9.56	1.24	0.52	2.92	0.63
		ILT	8.16	Ref			
FTT and ILT	*An. arabiensis*	FTT	13.64	1.36	0.73	2.56	0.33
		ILT	10.00	Ref			
	*An. funestus*	FTT	6.88	0.93	0.48	1.77	0.81
		ILT	7.96	Ref			
	*An. coustani*	FTT	7.12	0.79	0.32	1.91	0.59
		ILT	7.52	Ref			
HDT and ILT	*An. arabiensis*	HDT	10.24	1.25	0.65	2.40	0.50
		ILT	8.44	Ref			
	*An. funestus*	HDT	1.76	0.29	0.13	0.64	0.002
		ILT	6.24	Ref			
	*An. coustani*	HDT	1.80	0.21	0.10	0.49	< 0.001
		ILT	7.68	Ref			
HLC and ILT	*An. arabiensis*	HLC	1.80	0.26	0.11	0.59	0.001
		ILT	6.92	Ref			
	*An. funestus*	HLC	1.48	0.25	0.09	0.65	0.005
		ILT	5.92	Ref			
	*An. coustani*	HLC	10.52	1.24	0.54	2.90	0.60
		ILT	7.16	Ref			
OLT and ILT	*An. arabiensis*	OLT	3.56	0.42	0.20	0.89	0.03
		ILT	8.60	Ref		1	1
	*An. funestus*	OLT	1.20	0.21	0.09	0.81	< 0.001
		ILT	5.76	Ref			
	*An. coustani*	OLT	23.00	3.00	1.67	5.39	< 0.001
		ILT	7.64	Ref			

The models included terms for collection methods and an interaction term. The risk ratios (RR) were generated by exponentiating the model coefficients

**Table 4:** Comparison of mean numbers of *An. funestus*, *An. arabiensis* and *An. coustani* collected outdoors and indoor between each of the outdoor trapping methods (MET, FTT, HDT, OLT, HLC) and ILT in Masogo village

Comparison	*Anopheles* species	Collection method	Mean	Risk ratio	Lower CL	Upper CL	*P*-values
MET and ILT	*An. arabiensis*	MET	0.28	0.18	0.06	0.48	0.001
		ILT	1.44	Ref			
	*An. funestus*	MET	0.12	0.14	0.03	0.54	0.005
		ILT	0.88	Ref			
	*An. coustani*	MET	0.08	2.00	0.18	22.06	0.57
		ILT	0.04	Ref			
FTT and ILT	*An. arabiensis*	FTT	2.60	1.87	0.94	3.72	0.07
		ILT	1.48	Ref			
	*An. funestus*	FTT	2.16	1.20	0.57	2.53	0.64
		ILT	1.80	Ref			
	*An. coustani*	FTT	0.24	3.00	0.58	15.39	0.19
		ILT	0.08	Ref			
HDT and ILT	*An. arabiensis*	HDT	0.68	0.50	0.23	1.08	0.08
		ILT	1.48	Ref			
	*An. funestus*	HDT	0.36	0.28	0.09	0.80	0.02
		ILT	1.36	Ref			
	*An. coustani*	HDT	0.00	0.00	0.00	Inf	1.00
		ILT	0.16	Ref			
HLC and ILT	*An. arabiensis*	HLC	0.52	0.45	0.17	1.20	0.11
		ILT	1.08	Ref			
	*An. funestus*	HLC	0.52	0.48	0.15	1.52	0.21
		ILT	1.08	Ref			
	*An. coustani*	HLC	0.32	4	0.80	20.10	0.09
		ILT	0.08	Ref			
OLT and ILT	*An. arabiensis*	OLT	0.92	0.74	0.30	1.84	0.52
		ILT	1.20	Ref			
	*An. funestus*	OLT	0.24	0.21	0.08	0.57	0.002
		ILT	1.16	Ref			
	*An. coustani*	OLT	0.92	5.75	1.79	18.46	0.003
		ILT	0.16	Ref			

The models included terms for collection methods and an interaction term. The risk ratios (RR) were generated by exponentiating the model coefficients

In Kakola Ombaka, three of the outdoor trapping methods, HLC, OLT and HDT, collected significantly fewer *An. funestus* than the paired indoor ILT collections. The only outdoor trapping method to collect similar numbers of *An. funestus* as the ILT was the FTT. For *An. arabiensis* there was generally no significant difference in catch size between indoor light traps and outdoor methods, with the exception of HLC and OLT in Kakola Ombaka where numbers were significantly lower in both HLC and OLT compared to ILT. For *An. coustani*, there was no difference in the catch sizes between MET, FTT and HLC outdoors compared to ILT indoors. However, HDT sampled significantly fewer *An. coustani* while OLT sampled significantly more compared to ILT. In Masogo there were no clear differences although densities were much lower. The only outdoor trapping method to catch a greater number of *An. coustani* compared to paired ILT was the OLT. For all other methods there was no significant difference compared to ILT, except for the HDT, which caught significantly lower numbers.

Comparison of catch sizes in pairs of indoor and outdoor CDC light traps in Kakola Ombaka showed that significantly lower numbers of *An. arabiensis* (RR = 0.42, 95% CI 0.20–0.89, *P* = 0.03) and *An. funestus* (RR = 0.21, 95% CI 0.09–0.81, *P* < 0.001) were collected in OLT compared to indoor ILT whereas significantly higher numbers of *An. coustani* (RR = 3.00, 95% CI 1.67–5.39, *P* < 0.001) were observed in OLT compared to ILT (Table 3). A similar trend was also recorded in Masogo with fewer *An. funestus* in OLT than ILT (RR = 0.21, 95% CI 0.08–0.57, *P* = 0.002) but more *An. coustani* captured in OLT than ILT (RR = 5.75, 95% CI 1.79–18.46, *P* = 0.003) (Table 4).

Significantly lower numbers of *An. arabiensis* (RR = 0.26, 95% CI 0.11–0.59, *P* = 0.001) and *An. funestus* (RR = 0.25, 95% CI 0.09—0.65, *P* = 0.005) were collected by HLC outdoors compared to ILT in Kakola Ombaka. There was no significant difference in the numbers of *An. coustani* collected by these two methods. In Masogo, there was no significant difference in the catch sizes between the two HLC and ILT for all the three *Anopheles* species. There was no significant difference in the numbers of *An. arabiensis*, *An. funestus* and *An. coustani* sampled outdoors by FTT compared to paired ILT collections in Kakola Ombaka or Masogo (*P* > 0.05).

### Additional non-Latin square indoor and outdoor HLC collections

**Fig. 5:**
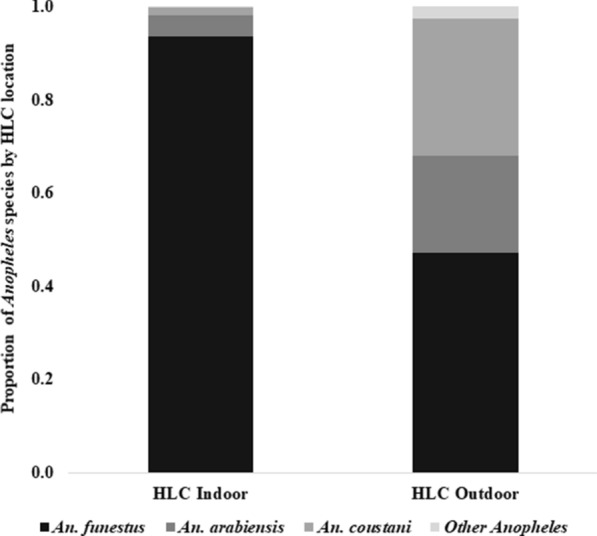
Relative species composition (proportions) of *Anopheles* mosquitoes from HLC indoor and outdoor from Kakola Ombaka village, western Kenya

A total of 1689 female *Anopheles* mosquitoes were collected by HLC indoors and outdoors at five trapping locations over 5 nights. Of the total collections, 1492 (88.3%) were *An. funestus*, 109 (6.5%) *An. arabiensis*, 81 (4.8%) *An. coustani* and 7 (0.4%) other species of *Anopheles*. From indoor HLC, *An. funestus* was the predominant species, accounting for 93.4%, while *An. arabiensis* was 4.6% and *An. coustani* 1.7%. Similarly, from outdoor HLC, *An. funestus* accounted for 47.1%, while *An. arabiensis* was 20.9%, *An. coustani* 29.3% and other *Anopheles* 2.6% (Fig. 5).

**Fig. 6:**
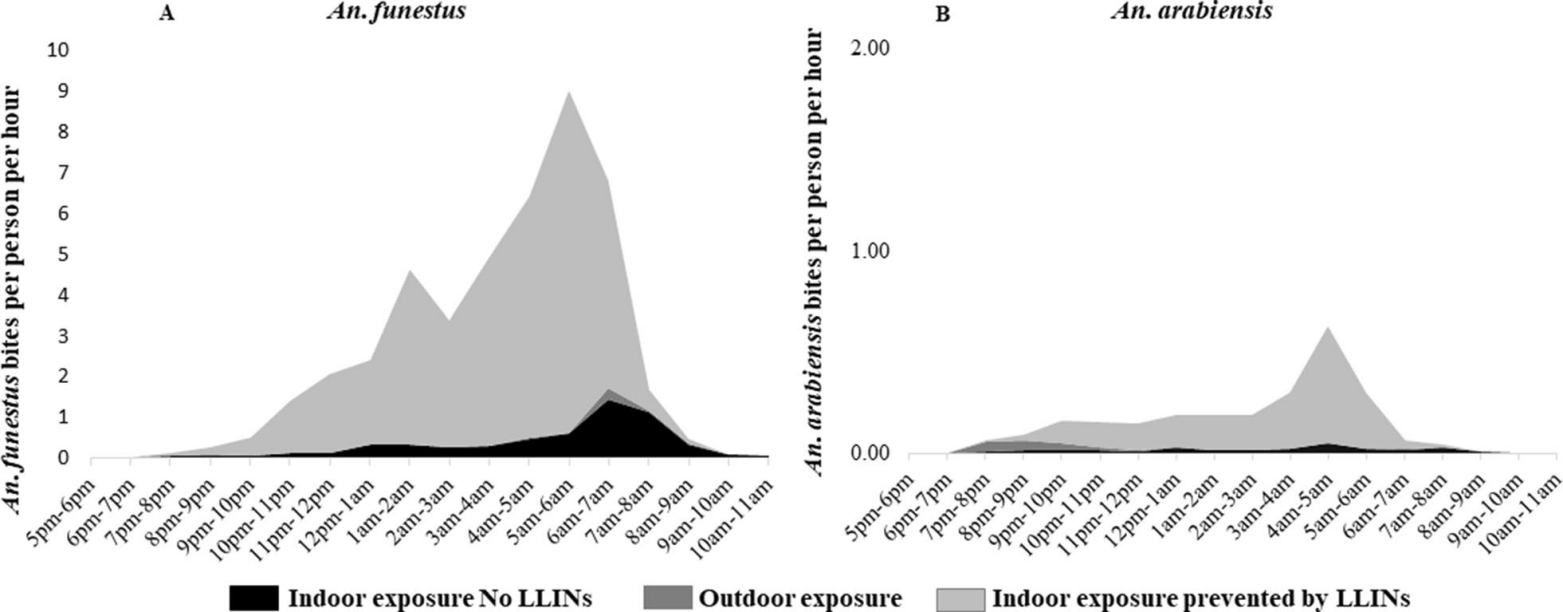
Profiles of biting by *An. funestus* (**A**) and *An. arabiensis* (**B**) experienced by the human population in Kakola Ombaka village, westen Kenya. The black area represents exposure that occurs outdoors, the red represents exposure that occurs indoors not prevented by LLINs and the grey represents exposure prevented by LLINs

We estimated the risk of human exposure to mosquito bites at one of the study villages, Kakola Ombaka. Figure 6 shows the number of bites per person per hour by *An. funestus* and *An. arabiensis*, adjusted by location of members of the household over the collection period to demonstrate the risk of exposure to mosquito bites. The risk of exposure to bites by *An. funestus* was highest indoors with peak at approximately nine bites per person occurring between 5:00 a.m. and 6:00 a.m., corresponding to the time when most people left their bedrooms (Additional file 2: Fig. S1) and were no longer protected by ITNs. Outdoor exposure to bites by *An. funestus* was mostly low with a peak of approximately two bites per person per night occurring between 6:00 a.m. and 7:00 a.m. Exposure occurring indoors in the living room while unprotected by ITNs was estimated to be less than a single bite per person per hour for most of the collection period with a peak risk of exposure occurring between 6:00 a.m. and 8:00 a.m. Extended exposure to *An. funestus* bites, albeit at low rates, was observed indoors until 11:00 a.m. when collections ceased (Fig. 6A).

The risk of exposure to bites by *An. arabiensis* was low compared to that of *An. funestus*, with a peak of less than a single bite per person per hour. Like biting by *An. funestus,* the peak biting by *An. arabiensis* occurred between 5:00 a.m. and 6:00 a.m., corresponding to the time when most people left their bedroom. Risk of exposure to bites by *An. arabiensis* outdoors was negligible (Fig. 6B).

An assessment of the location of members of the households showed, on average, 78% of individuals were outdoors between 5:00 p.m and 8:00 p.m. The proportion of people outdoors decreased steadily with a proportionate increase in numbers indoor over time. The proportion of people indoors, in the bedroom area was on average 83% between 8:00 p.m. and 6:00 a.m. and this decreased steadily between 6:00 a.m. and 7:00 a.m. with an increase in the numbers outdoors over the same time. On average, 69% of the people were outdoors between 6:00 a.m. and 11:00 a.m. when collection ceased (Additional file 2: Fig. S1).

### *Plasmodium falciparum* sporozoite rates

A sub-sample of 1253 *Anopheles* from outdoor traps and 1989 from indoor CDC light traps were analysed for sporozoite infection. From outdoor collections, the overall sporozoite infection rate was 0.4% (5/1253) with infection rates of 1.8% (4/221) in *An. funestus* and 0.2% in (1/599) *An. arabiensis.* From indoor CDC light trap collections, the overall sporozoite positivity rate was 1.0% (19/1989) with infection rates of 3.2% (17/538) in *An. funestus* and 0.2% (2/961) in *An. arabiensis*. No other species tested positive for sporozoites except *An. funestus* and *An. arabiensis*.

## Discussion

This study demonstrated marked differences in *Anopheles* catch sizes by species in outdoor traps compared to HLC. HLC was considered the gold standard for this study and the prediction was that all other outdoor traps would collect fewer *Anopheles* than HLC as has been reported in several studies comparing CDC LT indoors with HLC [25]. However, in Kakola Ombaka, HLC captured the fewest *An. arabiensis*, with all other outdoor traps catching greater densities. The ideal alternative to the HLC either indoors or outdoors should collect mosquitoes in densities that are consistently proportional to the HLC through a range of transmission intensities with similar species compositions. In addition, factors such as cost, feasibility and scalability should be considered in selecting a collection method for operational surveillance [23].

The Furvela tent trap has been described as a simple and effective way of collecting outdoor host-seeking mosquitoes [26]. Similarly, we observed the trap to be a simple tool for sampling mosquitoes that is easy to set up while being comfortable and safe from biting for the person occupying the tent. If the objective is to catch the greatest number of malaria vectors outdoors, the FTT was the most effective in sampling both *An. funestus* and *An. arabiensis* outdoors in the two villages. The trap sampled approximately six times more *An. funestus* compared to HLC in Kakola Ombaka and two times more in Masogo, while the catch size for *An. arabiensis* was approximately eight and five times more than HLC in the two sites respectively. Both *An. funestus* and *An. arabiensis* were caught in the FTT at nearly equal proportions, with relatively low numbers of *An. coustani* collected in the trap. An important consideration is whether the FTT is truly collecting outdoor host-seeking mosquitoes or is merely a scale model of an indoor environment that traps mosquitoes attempting to enter the tent as they would enter a house. Charlwood et al. concluded that the FTT was truly collecting outdoor biting species in Tanzania and Mozambique, based on the greater diversity of species (such as *Mansonia* and *Coquillettidia* spp.) compared to ILT and the greater densities of endophilic species such as *An. funestus* by ILT [26]. In this study we observed FTT outdoors closely mirrored ILT in both abundance and *Anopheles* species composition, with no significant difference in *An. funestus*, *An. arabiensis* or *An. coustani* densities between FTT and ILT. In contrast, the ILT densities of *An. funestus* were greater than all other outdoor trapping methods, including HLC. From this we conclude that *An. funestus* in the study site were predominantly endophilic, but the FTT mimicked indoor collections, capturing otherwise endophilic mosquitoes as they attempted to enter the tent. In an earlier trap comparison study in western Kenya, the Ifakara tent trap (another tent trap not used in the current investigation) was observed to be the only trap with the numbers of *An. funestus* captured not significantly different from HLC indoor [23]. These observations suggest that tent traps such as FTT are not truly outdoor trapping tools but mimics of an indoor environment where the trapped mosquito species exhibit house entry traits.

CDC light traps are commonly installed indoors adjacent to an occupied bed net [31, 32]. Outdoor implementation is more challenging, especially in regions where people do not routinely sleep outdoors, including the current study site. Collectors were reluctant to sleep outdoors because of possible security risks from humans and nocturnal animals and the relatively cold weather. OLT was the only trap that sampled more *An. coustani* in both villages compared to HLC. We conclude that the high densities of *An. coustani* were partly due to the exophilic nature of this species based on higher densities compared to ILT and partly due to attraction to the light bulb based on the higher densities compared to other outdoor traps. Compared with ILT, the OLT better sampled exophilic and exophagic *An. arabiensis* and *An. coustani* whilst the converse was true for *An. funestus*. Therefore, we conclude that the outdoor CDC light trap was the most useful outdoor trap tested in terms of determining outdoor host-seeking preferences and densities of *Anopheles* species. The CDC light traps have been reported to be the most effective alternative to HLC [21–23] when implemented indoors. Our results demonstrate that implementation of CDC light traps outdoors is similarly an effective alternative to HLC outdoors. However, further investigation is needed to generate data on hourly biting rates compared between CDC light traps and HLC to effectively demonstrate peaks of mosquito biting and exposure.

The host decoy trap was effective in sampling *An. arabiensis* compared to both HLC and ILT. We have previously demonstrated HDT with cattle odour to be effective for collecting *An. arabiensis* outdoors in western Kenya, whereas using human odour was not particularly effective [40]. However, in the current study, the HDT trap baited with a human sampled six times more *An. arabiensis* than HLC. The HDT combines host odours, heat and visual stimuli to simulate a host [29, 40], which provides the basis of sampling outdoor host-seeking mosquitoes with a potential of replacing HLC [29]. The trap however requires optimization of the heating system and odour source to ensure consistency and enable scalability and ease of application.

The MET showed promise as an outdoor mosquito trap, with comparable sampling sensitivity to HLC for *An. funestus* and *An. coustani* and higher sensitivity in sampling of *An. arabiensis* at relatively high mosquito densities in Kakola Ombaka. A study in the Kilombero Valley, southern Tanzania, observed the MET to achieve over 50% sampling sensitivity relative to HLC [41]. A separate study in Dar es Salaam investigating an improved prototype of MET observed the trap to be a highly sensitive tool that accurately quantifies epidemiologically relevant metrics of mosquito-biting densities, behaviours and human exposure distribution [42]. Elsewhere, in Burkina Faso, MET was observed to be less sensitive relative to HLC; however, the density of *An. gambiae* (*s.l.*) in MET was highly correlated with HLC [43]. We observed MET to correlate well with HLC at relatively high vector numbers but poorly at low mosquito numbers. While a number of studies have reported MET as a safer alternative to HLC for surveillance of mosquitoes outdoors [41–44], we observed the trap to pose an unacceptable level of discomfort to the collectors who were required to sit with minimal body movement, as the trap is located around their legs throughout the collection period.

The poor performance of HLC relative to other trapping methods in sampling outdoor biting mosquitoes may reflect its well-known limitation that accuracy and catch size are reliant on the performance of collectors and performance is difficult to measure. Every effort was made to ensure the quality of HLC collections by working with trained collectors, providing flasks of coffee and conducting nightly supervision. We hypothesize that the numbers of *Anopheles* mosquitoes collected were lower in HLC because of the large densities of *Culex* in the area, which may have led HLC collectors to focus on the larger *Culex* mosquitoes but miss some of the *Anopheles*. The standardization of other trapping methods is a potential advantage over HLC, which is prone to unknown levels of variation. However, HLC remains the most suitable method for assessing hourly biting behaviour of mosquitoes.

There is a risk of malaria transmission and other infections from both indoor and outdoor mosquito bites. Even though sporozoite infections were highest in indoor collections by CDC light traps, sporozoite-infected mosquitoes were observed outdoors albeit at low proportions. These results confirm the possibility of malaria transmission outdoors away from the protection of indoor based vector control tools. While it is important to monitor outdoor biting of malaria vectors, the level of risk is dependent on human behaviour and the amount of time spent outdoors. In Bioko, despite sustained indoor interventions through LLINs and IRS, fears of high outdoor malaria risk in children proved unfounded as only 4% of children spent time outside between 22:00 and 05:00 [45]. The risk of *Anopheles* mosquito bites was estimated based on the location of the study population during HLCs. Human activity outdoors steadily increased from 05:00 a.m. corresponding to the period of increased mosquito biting by both *An. funestus* and *An. arabiensis*. These observations indicated that in western Kenya the greatest risk of exposure was indoors late at night but with some outdoor exposure to both species, particularly in the early morning. Consequently, monitoring of outdoor mosquito populations as vector interventions such as ITNs or IRS reduce indoor transmission is critical in understanding the dynamics in malaria transmission.

Like any other study, this evaluation had limitations that merit further investigation. The study was conducted only in the rainy season with peak mosquito density. Therefore, the effect of seasonality on the trapping efficacy of the outdoor mosquito collection methods was not measured. Consequently, further analysis was not conducted to determine if efficacy of the traps was density dependent or not. It may, therefore, be necessary to conduct similar evaluations in both wet and dry seasons and in different ecological settings to measure the trapping efficacy of the various traps in these different scenarios.

## Conclusion

Sampling of outdoor mosquito population is important in understanding changes in the risk of malaria transmission with increased indoor mosquito control efforts. However, data on longitudinal outdoor mosquito monitoring are usually limited because of lack of a safe, efficient, easily deployed outdoor sampling tool. We observed Furvela tent trap to be a simple and effective tool for sampling mosquitoes, easy to set and exposure free with a higher sensitivity relative to HLC in sampling major *Anopheles* species in western Kenya. However, the trap closely mirrored indoor CDC light trap in mosquito indices and therefore may be more of an indoor mimic than a true outdoor collection tool. Outdoor human-baited CDC light trap on the other had is relatively simple to use and easily scalable and a suitable alternative to HLC with potential for collection of hourly mosquito data. The host decoy trap also demonstrated a potential for sampling outdoor host-seeking mosquitoes with a possibility of replacing HLC. However, the trap needs improvement regarding heating, odour sources and mosquito trapping mechanism to enable scalability. Finally, the MET showed variability in sensitivity relative to HLC at high and low vector densities and may not be easily scalable for routine mosquito collections. Based on these observations, the outdoor light trap is the most appropriate tool currently available for routine assessment of outdoor biting and malaria transmission risk in this setting.

## Supplementary Information

**Additional file 1: Table S1.** Schedule showing the rotation of volunteer sleepers and outdoor trapping methods by location (H1–H5) using a non-random Latin square rotation. **Table S2.** A pairwise comparison of means of different *Anopheles* species between collection methods in Kakola Ombaka. **Table S3.** A pairwise comparison of means of different *Anopheles* species between collection methods in Masogo.

**Additional file 2: Figure S1.** Proportion of individuals at different locations (outdoor, living room and bedroom) per collection hour.

## Data Availability

All data generated or analyzed during this study are included in this published article and its additional files.

## Abbreviations

HLC: Human landing catch
CDC-LT: CDC light trap
FTT: Furvela tent trap
MET: Mosquito electrocuting trap
HDT: Human decoy trap

## References

[1] Dekker T, Takken W, Braks MA (2001). Innate preference for host-odor blends modulates degree of anthropophagy of *Anopheles gambiae* sensu lato (Diptera: Culicidae). J Med Entomol.

[2] WHO. World Malaria Report. Geneva: Word Health Organization; 2019.

[3] Bhatt S (2015). The effect of malaria control on *Plasmodium falciparum* in Africa between 2000 and 2015. Nature.

[4] Bayoh MN (2010). *Anopheles gambiae*: historical population decline associated with regional distribution of insecticide-treated bed nets in western Nyanza Province Kenya. Malar J.

[5] McCann RS (2014). Reemergence of *Anopheles funestus* as a vector of *Plasmodium falciparum* in western Kenya after long-term implementation of insecticide-treated bed nets. Am J Trop Med Hyg.

[6] Musiime AK (2019). Impact of vector control interventions on malaria transmission intensity, outdoor vector biting rates and Anopheles mosquito species composition in Tororo, Uganda. Malar J.

[7] Abong'o B (2020). Impact of indoor residual spraying with pirimiphos-methyl (Actellic 300CS) on entomological indicators of transmission and malaria case burden in Migori County, western Kenya. Sci Rep.

[8] Ndenga BA (2016). Malaria vectors and their blood-meal sources in an area of high bed net ownership in the western Kenya highlands. Malar J.

[9] Ototo EN (2015). Surveillance of malaria vector population density and biting behaviour in western Kenya. Malar J.

[10] Killeen GF (2014). Characterizing, controlling and eliminating residual malaria transmission. Malar J.

[11] Degefa T (2017). Indoor and outdoor malaria vector surveillance in western Kenya: implications for better understanding of residual transmission. Malar J.

[12] Sougoufara S, Ottih EC, Tripet F (2020). The need for new vector control approaches targeting outdoor biting Anopheline malaria vector communities. Parasit Vectors.

[13] Killeen GF (2016). Most outdoor malaria transmission by behaviourally-resistant Anopheles arabiensis is mediated by mosquitoes that have previously been inside houses. Malar J.

[14] VaIe GA, Torr SJ (2015). Surveillance and sampling of disease vectors. Rev Sci Tech.

[15] Service MW. Mosquito ecology: field sampling methods, 2nd ed. London: Elsevier; 1993. p 988.

[16] Lindsay SW (1993). Variation in attractiveness of human subjects to malaria mosquitoes (Diptera: Culicidae) in The Gambia. J Med Entomol.

[17] Mukabana WR (2002). Host-specific cues cause differential attractiveness of Kenyan men to the African malaria vector *Anopheles gambiae*. Malar J.

[18] Ndebele P, Musesengwa R (2012). View point: Ethical dilemmas in malaria vector research in Africa: making the difficult choice between mosquito, science and humans. Malawi Med J.

[19] Achee NL (2015). Considerations for the use of human participants in vector biology research: a tool for investigators and regulators. Vector-Borne Zoon Dis.

[20] Gimnig JE (2013). Incidence of malaria among mosquito collectors conducting human landing catches in western Kenya. Am J Trop Med Hyg.

[21] Onyango SA (2013). Monitoring malaria vector control interventions: effectiveness of five different adult mosquito sampling methods. J Med Entomol.

[22] Sikaala CH (2013). Evaluation of alternative mosquito sampling methods for malaria vectors in Lowland South-East Zambia. Parasit Vectors.

[23] Wong J (2013). Standardizing operational vector sampling techniques for measuring malaria transmission intensity: evaluation of six mosquito collection methods in western Kenya. Malar J.

[24] Mathenge, E.M., et al., *Comparative performance of the Mbita trap, CDC light trap and the human landing catch in the sampling of Anopheles arabiensis, An. funestus and culicine species in a rice irrigation in western Kenya.* Malar J, 2005. **4**: 7. 10.1186/1475-2875-4-7 PMC548676 15667666

[25] Lines J (1991). Monitoring human-biting mosquitoes (Diptera: Culicidae) in Tanzania with light-traps hung beside mosquito nets. Bull Entomol Res.

[26] Charlwood JD, et al. The Furvela tent-trap Mk 1.1 for the collection of outdoor biting mosquitoes*.* PeerJ. 2017;5: e3848. 10.7717/peerj.3848 PMC5694212 29158961

[27] Charlwood JD (2012). Feeding frequency and survival of Anopheles gambiae in a rice-growing area in Ghana. Med Vet Entomol.

[28] Charlwood JD (2016). Effects of the spatial repellent metofluthrin on landing rates of outdoor biting anophelines in Cambodia Southeast Asia. Med Vet Entomol.

[29] Hawkes FM (2017). Exploiting Anopheles responses to thermal, odour and visual stimuli to improve surveillance and control of malaria. Sci Rep.

[30] Vale GA. New field methods for studying the responses of tsetse flies (Diptera, Glossinidae) to hosts*.* Bull Entomol Res, 1974; 64:199–208.

[31] Mboera LE (1998). Short report: influence of centers for disease control light trap position, relative to a human-baited bed net, on catches of *Anopheles gambiae* and *Culex quinquefasciatus* in Tanzania. Am J Trop Med Hyg.

[32] Costantini C (1998). Realationship to bitting colletions and influence of light and bednet in CDC light-trap catches of west Africa malaria vectors. Bull Entomol Res.

[33] Mboera LE (2005). Sampling techniques for adult Afrotropical malaria vectors and their reliability in the estimation of entomological inoculation rate. Tanzan Health Res Bull.

[34] Gillies MT, Coetzee M. A supplement to the anophelinae of Africa South of the Sahara (Sfrotropical region). 1987, Johannesburg: South African Institute for Medical Research.

[35] MR4, Methods in Anopheles Research, 2015 ed.CDC Atlanta, Gorgia, USA. 2007.

[36] Wirtz RA (1987). Comparative testing of monoclonal antibodies against *Plasmodium falciparum* sporozoites for ELISA development. Bull World Health Organ.

[37] Scott JA, Brogdon WG, Collins FH (1993). Identification of single specimens of the *Anopheles gambiae* complex by the polymerase chain reaction. Am J Trop Med Hyg.

[38] Koekemoer LL (2002). A cocktail polymerase chain reaction assay to identify members of the *Anopheles funestus* (Diptera: Culicidae) group. Am J Trop Med Hyg.

[39] Fournier DA (2012). AD Model {B}uilder: using automatic differentiation for statistical inference on highly parameterized complex nonlinear models. Optimiz Methods Softw.

[40] Abong'o B (2018). Host Decoy Trap (HDT) with cattle odour is highly effective for collection of exophagic malaria vectors. Parasit Vectors.

[41] Maliti DV (2015). Development and evaluation of mosquito-electrocuting traps as alternatives to the human landing catch technique for sampling host-seeking malaria vectors. Malar J.

[42] Govella NJ (2016). An improved mosquito electrocuting trap that safely reproduces epidemiologically relevant metrics of mosquito human-feeding behaviours as determined by human landing catch. Malar J.

[43] Sanou A (2019). Evaluation of mosquito electrocuting traps as a safe alternative to the human landing catch for measuring human exposure to malaria vectors in Burkina Faso. Malar J.

[44] Meza FC (2019). Mosquito electrocuting traps for directly measuring biting rates and host-preferences of Anopheles arabiensis and Anopheles funestus outdoors. Malar J.

[45] Bradley J (2012). Increased risks of malaria due to limited residual life of insecticide and outdoor biting versus protection by combined use of nets and indoor residual spraying on Bioko Island Equatorial Guinea. Malar J.

