# Supplementary material for: Comparison of four outdoor mosquito trapping methods as potential replacements for human landing catches in western Kenya
Source: Parasit Vectors. 2021 Jun 12;14:320. doi: 10.1186/s13071-021-04794-3 (PMC8196510; doi:10.1186/s13071-021-04794-3)
Supplement: Supplementary file 1 — Additional file 1: Table S1. Schedule showing the rotation of volunteer sleepers and outdoor trapping methods by location (H1–H5) using a non-random Latin square rotation. Table S2. A pairwise comparison of means of different Anopheles species between collection methods in Kakola Ombaka. Table S3. A pairwise comparison of means of different Anopheles species between collection methods in Masogo. [file 13071_2021_4794_MOESM1_ESM.doc]

**Supplemental tables**

Supplemental Table 1: Schedule showing the rotation of volunteer sleepers and outdoor trapping methods by location (H1-H5) using a non-random Latin square rotation.

|  | H1 | H2 | H3 | H4 | H5 |
| --- | --- | --- | --- | --- | --- |
| Night1 | HLC 1  Sleeper A | CDC-LT 1  Sleeper B | FTT 1  Sleeper C | MET 1  Sleeper D | HDT 1  Sleeper E |
| Night2 | FTT 1  Sleeper A | HDT 1  Sleeper B | HLC 1  Sleeper C | CDC-LT 1  Sleeper D | MET 1  Sleeper E |
| Night3 | HDT 1  Sleeper A | FTT 1  Sleeper B | MET 1  Sleeper C | HLC 1  Sleeper D | CDC-LT 1  Sleeper E |
| Night4 | CDC-LT 1  Sleeper A | MET 1  Sleeper B | HDT 1  Sleeper C | FTT 1  Sleeper D | HLC 1  Sleeper E |
| Night5 | MET 1  Sleeper A | HLC 1  Sleeper B | CDC-LT 1  Sleeper C | HDT 1  Sleeper D | FTT 1  Sleeper E |
| Break for weekend | | | | | |
| Night6 | HLC 1  Sleeper E | CDC-LT 1  Sleeper A | FTT 1  Sleeper B | MET 1  Sleeper C | HDT 1  Sleeper D |
| Night7 | FTT 1  Sleeper E | HDT 1  Sleeper A | HLC 1  Sleeper B | CDC-LT 1  Sleeper C | MET 1  Sleeper D |
| Night8 | HDT 1  Sleeper E | FTT 1  Sleeper A | MET 1  Sleeper B | HLC 1  Sleeper C | CDC-LT 1  Sleeper D |
| Night9 | CDC-LT 1  Sleeper E | MET 1  Sleeper A | HDT 1  Sleeper B | FTT 1  Sleeper C | HLC 1  Sleeper D |
| Night10 | MET 1  Sleeper E | HLC 1  Sleeper A | CDC-LT 1  Sleeper B | HDT 1  Sleeper C | FTT 1  Sleeper D |
| Break for weekend | | | | | |
| Night11 | HLC 1  Sleeper D | CDC-LT 1  Sleeper E | FTT 1  Sleeper A | MET 1  Sleeper B | HDT 1  Sleeper C |
| Night12 | FTT 1  Sleeper D | HDT 1  Sleeper E | HLC 1  Sleeper A | CDC-LT 1  Sleeper B | MET 1  Sleeper C |
| Night13 | HDT 1  Sleeper D | FTT 1  Sleeper E | MET 1  Sleeper A | HLC 1  Sleeper B | CDC-LT 1  Sleeper C |
| Night14 | CDC-LT 1  Sleeper D | MET 1  Sleeper E | HDT 1  Sleeper A | FTT 1  Sleeper B | HLC 1  Sleeper C |
| Night15 | MET 1  Sleeper D | HLC 1  Sleeper E | CDC-LT 1  Sleeper A | HDT 1  Sleeper B | FTT 1  Sleeper C |
| Break for weekend | | | | | |
| Night16 | HLC 1  Sleeper C | CDC-LT 1  Sleeper D | FTT 1  Sleeper E | MET 1  Sleeper A | HDT 1  Sleeper B |
| Night17 | FTT 1  Sleeper C | HDT 1  Sleeper D | HLC 1  Sleeper E | CDC-LT 1  Sleeper A | MET 1  Sleeper B |
| Night18 | HDT 1  Sleeper C | FTT 1  Sleeper D | MET 1  Sleeper E | HLC 1  Sleeper A | CDC-LT 1  Sleeper B |
| Night19 | CDC-LT 1  Sleeper C | MET 1  Sleeper D | HDT 1  Sleeper E | FTT 1  Sleeper A | HLC 1  Sleeper B |
| Night20 | MET 1  Sleeper C | HLC 1  Sleeper D | CDC-LT 1  Sleeper E | HDT 1  Sleeper A | FTT 1  Sleeper B |
| Break for weekend | | | | | |
| Night21 | HLC 1  Sleeper B | CDC-LT 1  Sleeper C | FTT 1  Sleeper D | MET 1  Sleeper E | HDT 1  Sleeper A |
| Night22 | FTT 1  Sleeper B | HDT 1  Sleeper C | HLC 1  Sleeper D | CDC-LT 1  Sleeper E | MET 1  Sleeper A |
| Night23 | HDT 1  Sleeper B | FTT 1  Sleeper C | MET 1  Sleeper D | HLC 1  Sleeper E | CDC-LT 1  Sleeper A |
| Night24 | CDC-LT 1  Sleeper B | MET 1  Sleeper C | HDT 1  Sleeper D | FTT 1  Sleeper E | HLC 1  Sleeper A |
| Night25 | MET 1  Sleeper B | HLC 1  Sleeper C | CDC-LT 1  Sleeper D | HDT 1  Sleeper E | FTT 1  Sleeper A |

Supplemental Table 2: A pairwise comparison of means of different *Anopheles* species between collection methods in Kakola Ombaka. (This could possibly go to the supplementary information)

| *Anopheles* species | Collection method | Difference in mean | Lower CL | Upper  CL | P-values adjusted |
| --- | --- | --- | --- | --- | --- |
| *An. coustani* | FTT-HDT | 5.32 | -5.29 | 15.93 | 0.72 |
|  | ILT-HDT | 5.83 | -2.39 | 14.05 | 0.32 |
|  | ECG-HDT | 7.76 | -2.85 | 18.37 | 0.29 |
|  | HLC-HDT | 8.72 | -1.89 | 19.33 | 0.17 |
|  | OLT-HDT | 21.20 | 10.58 | 31.81 | <0.0001 |
|  | ILT-FTT | 0.51 | -7.71 | 8.73 | 1.00 |
|  | ECG-FTT | 2.44 | -8.17 | 13.05 | 0.99 |
|  | HLC-FTT | 3.40 | -7.21 | 14.01 | 0.94 |
|  | OLT-FTT | 15.88 | 5.27 | 26.49 | 0.0004 |
|  | ECG-ILT | 1.92 | -6.29 | 10.15 | 0.98 |
|  | HLC-ILT | 2.89 | -5.33 | 11.11 | 0.91 |
|  | OLT-ILT | 15.37 | 7.15 | 23.59 | <0.0001 |
|  | HLC-ECG | 0.96 | -9.65 | 11.57 | 1.00 |
|  | OLT-ECG | 13.44 | 2.83 | 24.05 | 0.004 |
|  | OLT-HLC | 12.48 | 1.87 | 23.09 | 0.01 |
| *An. funestus* | HLC-OLT | 0.28 | -528 | 5.84 | 1.00 |
|  | HDT-OLT | 0.56 | -5.00 | 6.12 | 1.00 |
|  | ECG-OLT | 1.48 | -4.08 | 7.04 | 0.97 |
|  | ILT-OLT | 5.03 | 0.73 | 9.34 | 0.01 |
|  | FTT-OLT | 5.68 | 0.12 | 11.24 | 0.04 |
|  | HDT-HLC | 0.28 | -5.28 | 5.84 | 1.00 |
|  | ECG-HLC | 1.20 | -4.36 | 6.76 | 0.99 |
|  | ILT-HLC | 4.75 | 0.45 | 9.06 | 0.02 |
|  | FTT-HLC | 5.40 | -0.16 | 10.96 | 0.06 |
|  | ECG-HDT | 0.92 | -4.64 | 6.48 | 1.00 |
|  | ILT-HDT | 4.47 | 0.16 | 8.78 | 0.04 |
|  | FTT-HDT | 5.12 | -0.44 | 10.68 | 0.09 |
|  | ILT-ECG | 3.55 | -0.75 | 7.86 | 0.17 |
|  | FTT-ECG | 4.20 | -1.36 | 9.70 | 0.26 |
|  | FTT-ILT | 0.65 | -3.66 | 4.95 | 1.00 |
| *An. arabiensis* | OLT-HLC | 1.76 | -9.59 | 13.11 | 1.00 |
|  | ECG-HLC | 6.60 | -4.75 | 17.95 | 0.55 |
|  | ILT-HLC | 7.61 | -1.18 | 16.40 | 0.13 |
|  | HDT-HLC | 8.44 | -2.91 | 19.79 | 0.27 |
|  | FTT-HLC | 11.84 | 0.49 | 23.19 | 0.04 |
|  | ECG-OLT | 4.84 | -6.51 | 16.19 | 0.82 |
|  | ILT-OLT | 5.85 | -2.94 | 14.63 | 0.4 |
|  | HDT-OLT | 6.68 | -4.67 | 18.03 | 0.54 |
|  | FTT-OLT | 10.08 | -1.26 | 21.43 | 0.11 |
|  | ILT-ECG | 1.01 | -7.78 | 9.80 | 1.00 |
|  | HDT-ECG | 1.84 | -9.51 | 13.19 | 1.00 |
|  | FTT-ECG | 5.24 | -6.11 | 16.59 | 0.77 |
|  | HDT-ILT | 0.83 | -7.96 | 9.62 | 1.00 |
|  | FTT-ILT | 4.23 | -4.56 | 13.02 | 0.74 |
|  | FTT-HDT | 3.40 | -7.94 | 14.75 | 0.96 |

Supplemental Table 3: A pairwise comparison of means of different *Anopheles* species between collection methods in Masogo. (This could possibly go to the supplementary information)

| *Anopheles* species | Collection Method | Difference in mean | Lower  CL | Upper CL | P-value  adjusted |
| --- | --- | --- | --- | --- | --- |
| *An. coustani* | ECG-HDT | 0.08 | -0.42 | 0.58 | 0.99 |
|  | FTT-HDT | 0.24 | -0.26 | 0.74 | 0.68 |
|  | HLC-HDT | 0.32 | -0.18 | 0.82 | 0.4 |
|  | OLT-HDT | 0.92 | 0.42 | 1.42 | <0.001 |
|  | FTT-ECG | 0.16 | -0.34 | 0.66 | 0.9 |
|  | HLC-ECG | 0.24 | -0.26 | 0.74 | 0.68 |
|  | OLT-ECG | 0.84 | 0.34 | 1.34 | <0.001 |
|  | HLC-FTT | 0.08 | -0.42 | 0.58 | 0.99 |
|  | OLT-FTT | 0.68 | 0.17 | 1.18 | 0.003 |
|  | OLT-HLC | 0.60 | 0.10 | 1.10 | 0.01 |
| *An. funestus* | OLT-ECG | 0.12 | -1.27 | 1.51 | 1 |
|  | HDT-ECG | 0.24 | -1.15 | 1.62 | 1 |
|  | HLC-ECG | 0.40 | -0.99 | 1.79 | 0.96 |
|  | ILT-ECG | 1.14 | 0.06 | 2.21 | 0.03 |
|  | FTT-ECG | 2.04 | 0.65 | 3.43 | 0.0005 |
|  | HDT-OLT | 0.12 | -1.27 | 1.51 | 1 |
|  | HLC-OLT | 0.28 | -1.11 | 1.67 | 0.99 |
|  | ILT-OLT | 1.02 | -0.05 | 2.09 | 0.08 |
|  | FTT-OLT | 1.92 | 0.53 | 3.31 | 0.001 |
|  | HLC-HDT | 0.16 | -1.23 | 1.55 | 1 |
|  | ILT-HDT | 0.90 | -0.18 | 1.97 | 0.16 |
|  | FTT-HDT | 1.80 | 0.41 | 3.18 | 0.003 |
|  | ILT-HLC | 0.74 | -0.34 | 1.81 | 0.36 |
|  | FTT-HLC | 1.64 | 0.25 | 3.03 | 0.01 |
|  | FTT-ILT | 0.90 | -0.17 | 1.98 | 0.15 |
| *An. arabiensis* | CHLC-ECG | 0.24 | -1.39 | 1.87 | 1 |
|  | HDT-ECG | 0.04 | -0.99 | 2.27 | 0.87 |
|  | OLT-ECG | 0.64 | -0.99 | 2.27 | 0.86 |
|  | ILT-ECG | 1.06 | -0.20 | 2.32 | 0.16 |
|  | FTT-ECG | 2.32 | 0.69 | 3.95 | 0.001 |
|  | HDT-HLC | 0.16 | -1.47 | 1.79 | 1 |
|  | OLT-HLC | 0.40 | -1.23 | 2.03 | 0.98 |
|  | ILT-HLC | 0.82 | -0.44 | 2.08 | 0.43 |
|  | FTT-HLC | 2.08 | 0.45 | 3.71 | 0.004 |
|  | OLT-HDT | 0.24 | -1.38 | 1.87 | 1 |
|  | ILT-HDT | 0.66 | -0.60 | 1.92 | 0.67 |
|  | FTT-HDT | 1.92 | 0.29 | 3.55 | 0.01 |
|  | ILT-OLT | 0.42 | -0.84 | 1.68 | 0.93 |
|  | FTT-OLT | 1.68 | 0.05 | 3.31 | 0.03 |
|  | FTT-ILT | 1.26 | 0.00 | 2.52 | 0.05 |
