# Supplementary figures and images for: Comparison of four outdoor mosquito trapping methods as potential replacements for human landing catches in western Kenya
Source: Parasit Vectors. 2021 Jun 12;14:320. doi: 10.1186/s13071-021-04794-3 (PMC8196510; doi:10.1186/s13071-021-04794-3)

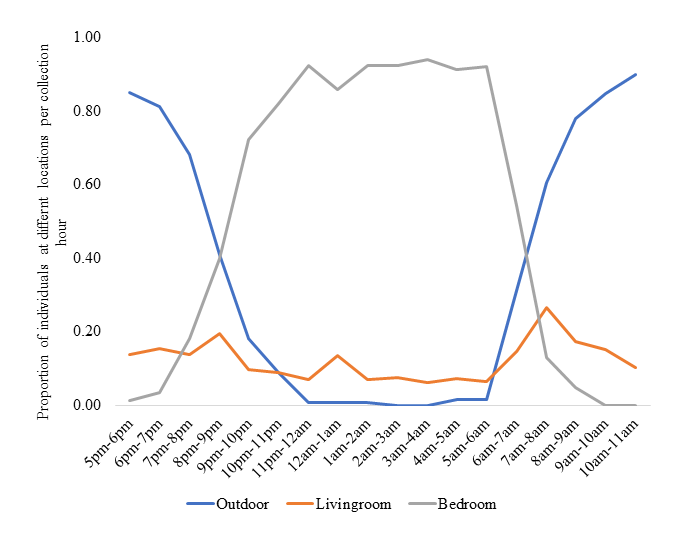

Supplement: Supplementary file 2 — Additional file 2: Figure S1. Proportion of individuals at different locations (outdoor, living room and bedroom) per collection hour. [file 13071_2021_4794_MOESM2_ESM.tif]
